# Sinusoidal Phase-Modulated Angle Interferometer for Angular Vibration Measurement

**DOI:** 10.3390/s21186295

**Published:** 2021-09-20

**Authors:** Xianfan Wang, Jianhua Yang, Meng Chen, Lijun Miao, Tengchao Huang

**Affiliations:** State Key Laboratory of Modern Optical Instrumentation, Zhejiang University, No. 38, Zheda Road, Hangzhou 310027, China; wangxianfan@zju.edu.cn (X.W.); Xxianhua@zju.edu.cn (J.Y.); chenmenghit@126.com (M.C.); huangtengchao@zju.edu.cn (T.H.)

**Keywords:** SPMAI, PGC demodulation, angular vibration calibration

## Abstract

Primary angular vibration calibration devices based on laser interferometers play a crucial role in evaluating the dynamic performance of inertial sensing devices. Here, we propose a sinusoidal phase-modulated angle interferometer (SPMAI) to realize angular vibration measurements over a frequency range of 1–1000 Hz, in which the sinusoidal measurement retro-reflector (SMR) and the phase generation carrier (PGC) demodulation algorithm are adopted to track the dynamic angle variation. A comprehensive theoretical analysis is presented to reveal the relationship between demodulation performance of the SPMAI and several factors, such as phase modulation depth, carrier phase delay and sampling frequency. Both the simulated and experimental results demonstrate that the proposed SPMAI can achieve an angular vibration measurement with amplitude of sub-arcsecond under given parameters. Using the proposed SPMAI, the frequency bandwidth of an interferometric fiber-optic gyroscope (IFOG) is successfully determined to be 848 Hz.

## 1. Introduction

Inertial sensing devices (ISDs), such as accelerometers or gyroscopes, have been widely used in various applications ranging from precision measurement, motion tracking and automatic piloting to missile guidance. To evaluate the dynamic performance of ISDs accurately, many types of angular-vibration-measurement approaches and instruments have been proposed. Referring to ISO 16063, there are several considerable approaches to realize angular vibration measurements, such as primary calibration by laser interferometry, primary calibration by the reciprocity method and calibration by comparison to a reference transducer [1]. Compared with other approaches, the primary calibration by laser interferometry has a higher measurement accuracy due to its direct traceability to length standard. The Physikalisch-Technische Bundesanstalt (PTB), in Germany, has adopted several types of laser interferometers to realize angular vibration calibrations, such as the Michelson grating interferometer and the Mach-Zehnder grating interferometer [2]. Later, the Changcheng Institute of Metrology and Measurement of China (CIMM) established a primary angular vibration calibration system based on a heterodyne interferometer and column grating and achieved an amplitude uncertainty of better than 1% over a frequency range of 0.1–200 Hz [3]. In addition, the Korea Research Institute of Standards and Science (KRISS) fabricated an angle-prism-based laser interferometer and its measurement uncertainty has been analyzed [4]. In 2012, PTB and KRISS carried out the bilateral comparison in primary angular vibration calibrations by using two angular accelerators and the measured expanded uncertainty values (including sensitivity and phase shift) within the angular vibration frequency range of 1 Hz–1000 Hz were presented [5]. It should be noted that these institutions generally employed homodyne laser interferometers or heterodyne laser interferometers. However, there exist several issues in practical applications. For example, in order to reduce DC noise, the homodyne laser interferometer has a large size and a complex signal-processing part [6]. On the other hand, the fabrication costs of the heterodyne laser interferometer still hinders its wide applications [7].

The sinusoidal phase-modulated laser interferometer (SPMI), first proposed in 1986, can be a good candidate to overcome the above-mentioned issues [8]. By modulating the reference of the SPMI, the measured information is loaded on a series of high-frequency carriers, which effectively enhances its resistance to DC noise. On the other hand, the optical composition of the SPMI is based on a conventional homodyne laser interferometer, the fabrication of which costs far less than that of a heterodyne laser interferometer. C. Zhang et al. used an SPMI to measure the angular displacement of an object with a measurement repeatability of 2.7 × 10^−7^ rad (0.05″) with the help of a Fabry–Perot plate [9]. In recent years, many researchers devoted their efforts to the study of SPMIs and the optical layout and demodulation algorithm of the SPMI have improved a lot. For example, L. Yan et al. used the SPMI to realize a nanometer displacement measurement by incorporating an additional triangular signal [10]. C. Ni et al. proposed a novel signal processing with ellipse fitting and correction method to compensate measurement errors of the SPMI [11].

To widen the applications of SPMIs, a sinusoidal measurement retro-reflector (SMR) is adopted, forming a novel sinusoidal phase-modulated angle interferometer (SPMAI). The SPMAI, as a primary angle measurement instrument, plays a crucial role in ISD calibration, precision manufacturing and attitude observation. The SMR and phase generation carrier (PGC) demodulation algorithm are used to convert the rotation angle into phase difference. In this paper, firstly, the optical configuration and measurement principle of the SPMAI are introduced. Through theoretical analysis and simulations, we reveal that relationships among the demodulated angle errors and several factors, such as modulation depth deviation and carrier phase delay, as well as sampling frequency. Furthermore, a measurement instrument based on SPMAI is designed and fabricated, with which the measured results can be obtained from measuring the interference signals using a data acquisition board (DAQ). Finally, with the help of a high-frequency angular vibration exciter, the SPMAI is used to measure angular vibrations within the frequency range of 1–1000 Hz and determine the frequency bandwidth of an IFOG.

## 2. Measurement Principles

**Figure 1:**
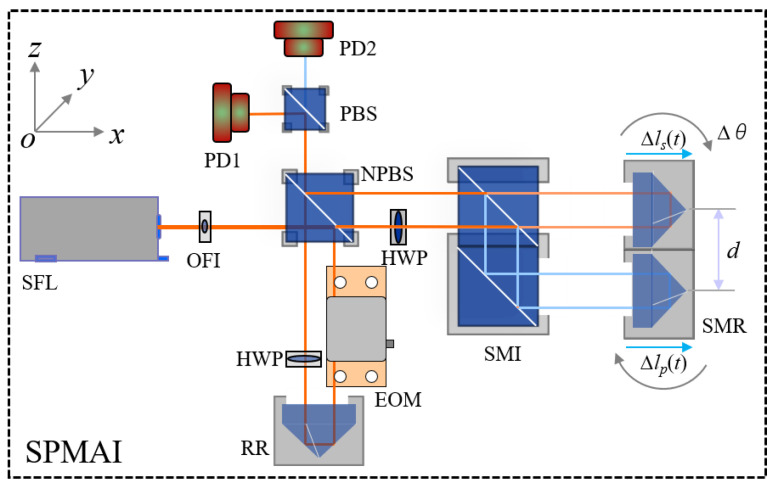
Schematic diagram of the SPMAI.

The optical configuration of the proposed SPMAI is shown as Figure 1. The SPMAI is based on a symmetrical Michelson-type interferometer. The single-frequency He–Ne laser (SFL) is used to emit a linearly polarized beam, which is separated into two parts after a non-polarizing beam splitter (NPBS), a reference beam (RB) and a measurement beam (MB). An optical Faraday isolator (OFI) is placed between the SFL and the NPBS. It should be noted that the use of the OFI not only can prevent any stray light from reflecting onto the laser, but can also rotate the polarization direction of the laser beam to direct it along the *y* axis. The RB is reflected by the NPBS and transmitted through an electro-optical phase modulator (EOM), a reference retro-reflector (RR) and a half-wave plate (HWP) in sequence. The EOM can modulate the phase of the laser beam under the added drive voltage *V_i_* and the modulated phase *φ_eom_* can expressed as *φ_eom_* = (*V_i_*/*V_π_*)*π*, where *V_π_* denotes half-wavelength voltage of the EOM. The MB is transmitted through the NPBS and further separated into two parts by a sinusoidal measurement interferometer (SMI) based on its polarization states, *s*-polarized MB (*s*-MB) and *p*-polarized MB (*p*-MB). Here, the SMI is constructed by two polarizing beam splitters (PBSs). Both *s*-MB and *p*-MB are incident on the two measurement retro-reflectors (MRs) of the SMR and their reflected beams are recombined at the NPBS. Furthermore, using a PBS, the *s*-polarized beam of RB and *s*-MB are reflected and their produced interference signals *I_s_* are detected by the photo-detector 1 (PD1). Similarly, the *p*-polarized beam of RB and *p*-MB are transmitted through the PBS and their interference signals *I_p_* are detected by the PD2. It should be noted that the SMR is constructed by two identical retro-reflectors. Compared with other counterparts (i.e., reflective mirror and right-angle prism), the retro-reflector can reflect the incident beam by 180° within a wider incident angle range. These two retro-reflectors are fixed by a designed mechanical structure and their separation distance is assumed as *d*. In our previous paper [12], the effect of the thickness difference and parallel error on measurement angle error has been analyzed. As the SMR is rotated, the optical path of the *s*-MB and *p*-MB is changed and, thus, *I_s_* and *I_p_* have a corresponding change. As the EOM is modulated by a sinusoidal voltage signal, *I_s_* and *I_p_* can be written as

(1) $$I_{s}=I_{s0}+I_{s1}\cos(c\cos2\pi f_{c}t+\frac{4\pi }{\lambda }\Delta l_{s}(t)),$$

(2) $$I_{p}=I_{p0}+I_{p1}\cos(c\cos2\pi f_{c}t+\frac{4\pi }{\lambda }\Delta l_{p}(t)),$$

where *I_s_*_0_ and *I_p_*_0_ denote the DC components, while *I_s_*_1_ and *I_p_*_1_ denote the AC amplitudes which are related to visibility of the measurement interference signals; *c* and *f_c_* denote the phase modulation depth and frequency, respectively; ∆*l_s_* and ∆*l_p_*, respectively, denote the optical path variation of the *s*-MB and *p*-MB as the SMR is rotated. Finally, the relationship between the rotary angle ∆*θ* and ∆*l_s_* and ∆*l_p_* can be written as

(3) $$\Delta \theta (t)=\arcsin(\frac{\left|\Delta l_{s}(t)\right|+\left|\Delta l_{p}(t)\right|}{d}).$$

Hence, the real-time angle values of the SMR can be obtained when ∆*l_s_* and ∆*l_p_* are determined.

**Figure 2:**
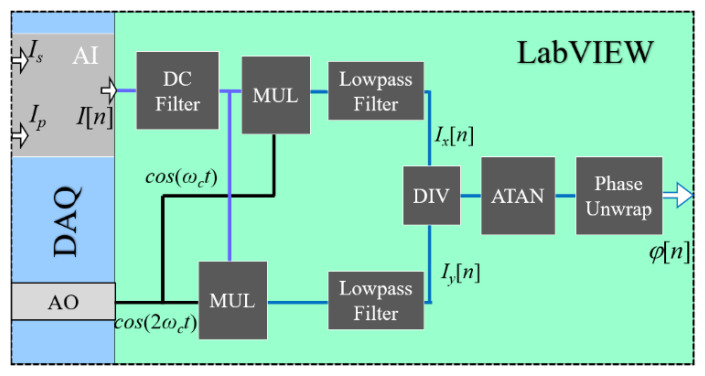
Schematic diagram of the PGC-Arctan demodulation algorithm.

In order to obtain the ∆*l_s_* and ∆*l_p_* in Equation (3), the PGC demodulation algorithm is adopted. The PGC demodulation technique, such as PGC-Arctan and PGC-DCM, has been widely used in optical interferometer due to its high sensitivity, large dynamic range and good linearity [13,14,15]. In the PGC demodulation process, the interference signals in Equations (1) and (2) are multiplied with the fundamental carrier and second-harmonic carrier and a pair of quadrature components are obtained. Here, the conventional PGC-Arctan demodulation algorithm based on the DAQ and LabVIEW platform is adopted and its operation principle is shown in Figure 2. Firstly, the measurement interference signals are detected by the PDs and two analog voltage signals are produced. By employing a DAQ, these two voltage signals are sampled and converted into digital signals (*I*[*n*]). Meanwhile, the DAQ can export modulation voltage signals with carrier frequency to the EOM. Furthermore, the LabVIEW program is used to obtain the phase change based on PGC-Arctan demodulation algorithm. Firstly, the digital voltage signals go through a DC filter to eliminate the DC component; then, they are multiplied with the fundamental carrier and second-harmonic carrier. After a low-pass filter, two quadrature components can be obtained, as follows:

(4) $$I_{x}[n]=GJ_{1}(c)\sin\varphi [n],$$

(5) $$I_{y}[n]=GJ_{2}(c)\cos\varphi [n],$$

where *J*_1_ and *J*_2_ denote first-order and second-order Bessel functions, *G* denotes the gain coefficient and *φ* denotes the phase variation related to rotation angle. According to the properties of the Bessel function, *J*_1_(*c*) is equal to *J*_2_(*c*), as *c* is 2.63 rad. Thus, *φ* can be easily calculated, based on the phase unwrapping algorithm, as

(6) $$\varphi [n]=\arctan(\frac{I_{x}[n]}{I_{y}[n]})+N\cdot 2\pi ,$$

where *N* is the count for the phase unwrapping in the PGC-Arctan demodulation algorithm.

## 3. Theoretical Analysis and Simulations

In ideal conditions, the real-time angle variation can be derived accurately by using the PGC-Arctan demodulation algorithm introduced above. However, there exist several factors influencing the measurement accuracy in the PGC-Arctan demodulation algorithm, such as the modulation depth deviation [16] and carrier phase delay [17], as well as the carrier frequency and sampling rate.

Here, we analyzed the relationship between the factors above and the measurement accuracy within an angular vibration frequency range of 1–1000 Hz. As the modulation depth deviation and carrier phase delay are considered, the measurement interference signals in Equations (1) and (2) can be rewritten as

(7) $$I_{s}^{\prime }=I_{s0}^{\prime }+I_{s1}^{\prime }\cos\left(c_{m}\cos\left(2\pi f_{c}t+\sigma \right)+\frac{4\pi }{\lambda }\Delta l_{s}(t)\right),$$

(8) $$I_{p}^{\prime }=I_{p0}^{\prime }+I_{p1}^{\prime }\cos\left(c_{m}\cos\left(2\pi f_{c}t+\sigma \right)+\frac{4\pi }{\lambda }\Delta l_{p}(t)\right),$$

where *c_m_* denotes the actual modulation depth, which is different from 2.63 rad, while *σ* denotes the carrier phase delay. Using the PGC-Arctan demodulation algorithm introduced above, the phase in Equation (6) can be written as [18]

(9) $$\varphi [n]=\varphi [n]+\frac{\nu -1}{2}\sin2\varphi [n],$$

where ν is related to *c_m_* and *θ*, which can be expressed as

(10) $$\nu =\frac{J_{1}(c_{m})\cos\sigma }{J_{2}(c_{m})\cos2\sigma }.$$

**Figure 3:**
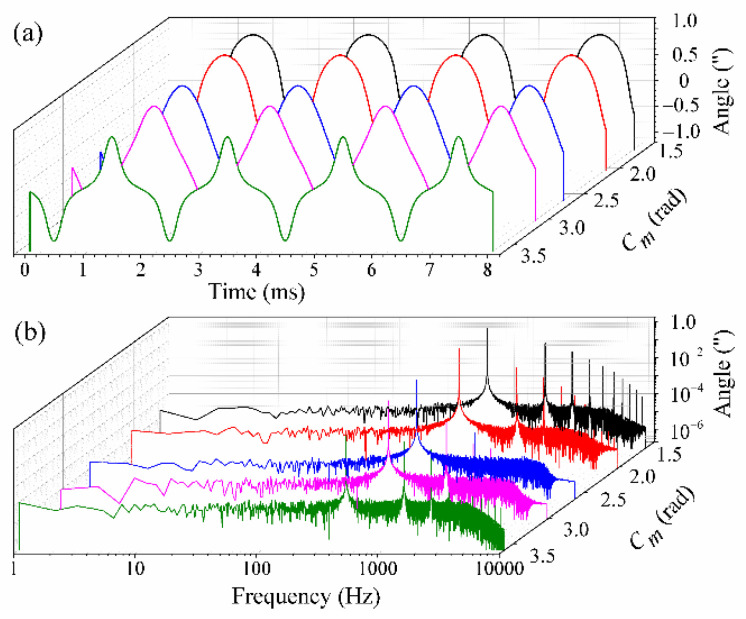
The simulated demodulation results in (**a**) time domain and (**b**) frequency domain as *c_m_* is increased from 1.63 rad to 3.63 rad.

It can be noted, from Equations (9) and (10), that the demodulation results cannot be influenced by the laser intensity disturbance; however, the existence of the modulation depth deviation and carrier phase delay would result in a demodulation error. Firstly, the relationship between the demodulation results and *c_m_* was investigated, as other factors were omitted. Here, the carrier modulating frequency and sampling rate were respectively assumed to be 10 kHz and 100 kHz. The amplitude and vibration frequency of the simulated signals were assumed to be 1″ and 500 Hz, respectively. In addition, the white Gaussian noise with an SNR of 50 dB was added to the simulated signals. As *c_m_* was increased from 1.63 rad to 3.63 rad with an arbitrary increment, the simulated results are shown in Figure 3. Figure 3a presents the demodulation results in the time domain. From Figure 3a, one can see that the waveform distortion becomes more obvious as *c_m_* deviates from 2.63 rad. Using the fast Fourier transform (FFT), the demodulation results in the frequency domain are obtained and shown in Figure 3b. From Figure 3b, one can see that there exist several high-frequency harmonic components when *c_m_* does not equal to 2.63 rad. Furthermore, the total harmonic distortion (THD) and the signal-to-noise and distortion ratio (SINAD) are used to evaluate the demodulation results obtained with the PGC-Arctan demodulation algorithm. It should be noted that THD refers to the ratio between the equivalent root-mean-square (RMS) amplitude of all harmonic frequencies and the amplitude of the fundamental frequency, while SINAD refers to the ratio of the power of the fundamental frequency to the sum of the powers of all noise and harmonic frequencies. Here, the amplitude, THD and SINAD of the demodulation results with different *c_m_* were calculated and the calculated results are shown in Table 1. From Table 1, one can find that the waveform distortion deteriorated increasingly as the difference between *c_m_* and 2.63 rad was increased, which is in accordance with the above theoretical analysis.

**Figure 4:**
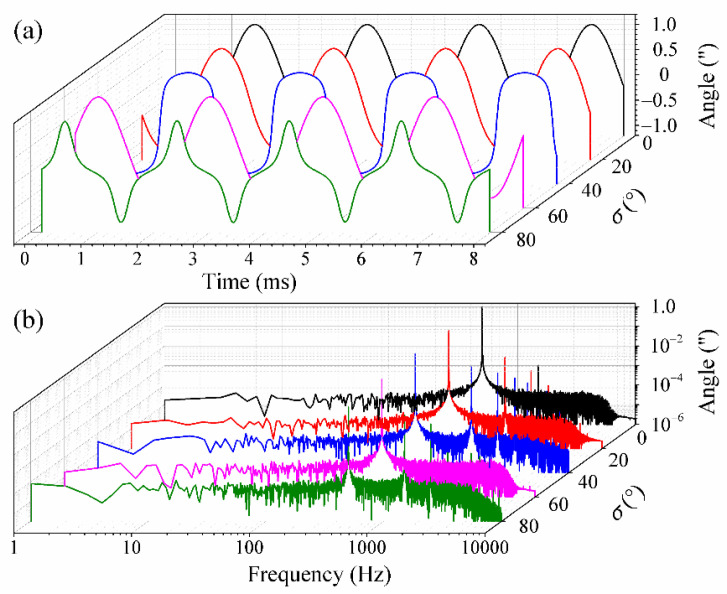
The simulated demodulation results in (**a**) time domain and (**b**) frequency domain as *σ* is increased from 0 to 80°.

Next, the dependence of the demodulation results on *σ* was investigated. The carrier modulating frequency, sampling rate and the amplitude and vibration frequency of the simulated signals were assumed to be the same as above. As *c_m_* was kept to 2.63 rad, the demodulation results for angular vibration of 500 Hz were obtained and the simulated results are shown in Figure 4. Figure 4a shows the demodulated waveform in time domain, while Figure 4b shows that for the frequency domain. From these, we can see that there exist several high-order harmonic frequencies when *σ* does not equal to 0 or 60°. Finally, the variations of the amplitude, THD and SINAD with different *σ* were calculated, as shown in Table 2. From Table 2, it can be seen that the amplitude values are kept steady, while the THD and SINAD change considerably with different *σ.* It should be noted that the *v* in Equation (10) equals to 1 when *σ* is chosen as 0 or 60° and the demodulation results have a THD of less than 2% and a SINAD of more than 50 dB. 

Moreover, the influence of the carrier frequency and sampling frequency on the demodulation results was also investigated. Here, the carrier frequency and sampling rate are denoted as *f_c_* and *F_s_*, respectively. The measured signals can be assumed to be *θ*(*t*) = *Dcos*(2π*f_s_t*), where *D* and *f_s_* denote the amplitude and frequency of the angular vibration, respectively. As the SMR is rotated around its central position, we obtain *l_s_*(*t*) = −*l_p_*(*t*) = 0.5*d* sin*θ*(*t*). The rotation angle of the SMR is so little that the relation between *l_s_*(*t*) or *l_p_*(*t*) and *θ*(*t*) can be approximated as *l_s_*(*t*) = −*l_p_*(*t*) = 0.5*d*·*θ*(*t*). Thus, the measurement interference signals in Equations (1) and (2) can be rewritten as

(11) $$I_{s}^{\prime \prime }=I_{s0}^{\prime \prime }+I_{s1}^{\prime \prime }\cos\left(c\cos2\pi f_{c}t+\frac{2\pi dD}{\lambda }\cos2\pi f_{s}t\right),$$

(12) $$I_{p}^{\prime \prime }=I_{p0}^{\prime \prime }+I_{p1}^{\prime \prime }\cos\left(c\cos2\pi f_{c}t-\frac{2\pi dD}{\lambda }\cos2\pi f_{s}t\right).$$

Using the PGC-Arctan demodulation algorithm introduced above, a pair of quadrature components can be obtained as

(13) $$I_{x}^{\prime \prime }=GJ_{1}(c)\sin\left(\frac{4\pi dD}{\lambda }\cos2\pi f_{s}t\right),$$

(14) $$I_{y}^{\prime \prime }=GJ_{2}(c)\cos\left(\frac{4\pi dD}{\lambda }\cos2\pi f_{s}t\right).$$

Using the Bessel function expansion, the sin factor and cos factor in Equations (13) and (14) can be written as

(15) $$\begin{array}{l} \sin\left(\frac{4\pi d}{\lambda }D\cos2\pi f_{s}t\right)=-2\sum_{m=1}^{\infty }{\left(-1\right)}^{m}\\ J_{2m-1}\left(\frac{4\pi dD}{\lambda }\right)\cos\left(\left(2m-1\right)2\pi f_{s}t\right) \end{array},$$

(16) $$\begin{array}{l} \cos\left(\frac{4\pi d}{\lambda }D\cos2\pi f_{s}t\right)=J_{0}\left(\frac{4\pi dD}{\lambda }\right)+\\ 2\sum_{m=1}^{\infty }\left({\left(-1\right)}^{m}J_{2m}(\frac{4\pi dD}{\lambda })\cos\left(4mf_{s}t\right)\right) \end{array}.$$

**Figure 5:**
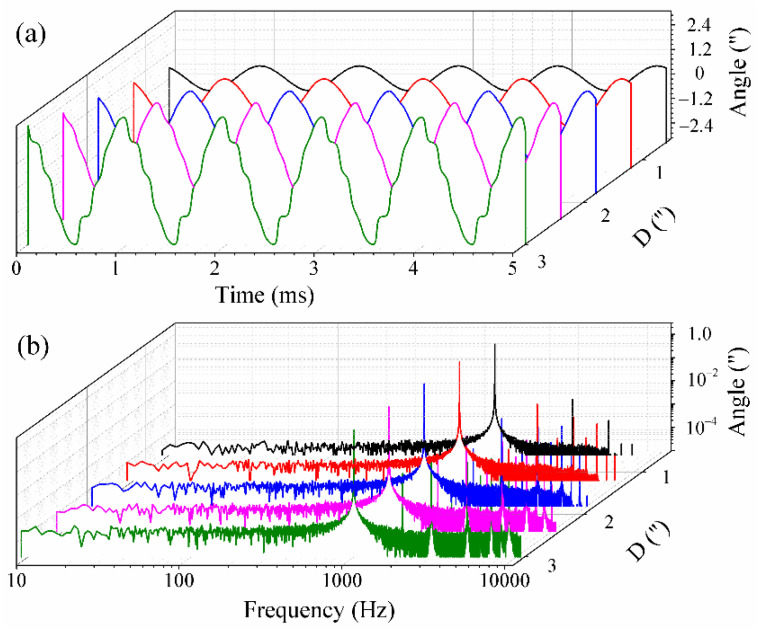
The simulated demodulation results in (**a**) time domain and (**b**) frequency domain as *D* is increased from 0.2″ to 3″.

**Figure 6:**
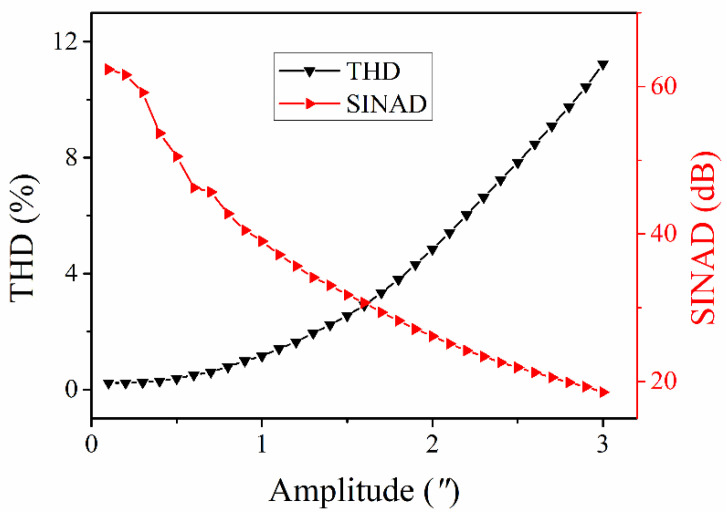
The calculated THD and SINAD of demodulation results with different *D* at 1 kHz.

From these two equations, the measured signals are expanded into a series of high-order harmonic frequencies, the amplitude values of which are dependent on the order of the Bessel function. It is well known that the value the Bessel function is decreased greatly with the order. In practical engineering applications, the harmonic frequency can be neglected if its amplitude is less than ten percent of the total amplitudes of the lower-order harmonic frequencies. For the PGC-Arctan demodulation algorithm used, the harmonic frequencies at orders of more than 3 were neglected; thus, the sampling frequency should meet *F_s_* > 5*f_c_*. On the other hand, the dynamic range of the PGC-Arctan demodulation algorithm can also be determined as *D* < *f_c_*/2*f_s_*. It should be noted that the performance of the used low-pass filters was assumed to be ideal; thus, their transition frequency band was not considered. Here, *f_s_*, *f_c_* and *F_s_* were respectively assumed to 1 kHz, 10 kHz and 100 kHz, while the amplitude values of the angular vibration were increased from 0.6″ to 3″ with an increment of 0.6″. The simulated demodulation results of the PGC-Arctan algorithm used are presented in Figure 5. Figure 5a shows the demodulation results in the time domain as the amplitude of the measured signals was increased from 0.6″ to 3″. From Figure 5a, one can see that the waveform distortion becomes obvious with the increase in *D*. Figure 5b shows the corresponding demodulation results in the frequency domain. The variations of the amplitude, THD and SINAD with different *D* were calculated, as shown in Table 3. From Table 3, one can see that the demodulated amplitude changed slightly at these chosen D values within 0.6″–3″. However, with the increase in *D*, the THD increased considerably and the SINAD decreases greatly, which demonstrates the demodulation capability is limited by *D* at a certain frequency. Furthermore, the dependence of the THD and the SINAD on *D* is presented in Figure 6. From Figure 6, one can see that the THD increased gradually as *D* was increased from 0 to 3″. On the other hand, there exists an inversely proportional relationship between the SINAD and *D*, which is consistent with the above theoretical analysis. In general, a SINAD of 30 dB can meet our angular vibration measurement, thus a dynamic range of more than 1.6″ can be provided at the frequency of 1 kHz.

## 4. Experiments and Results

**Figure 7:**
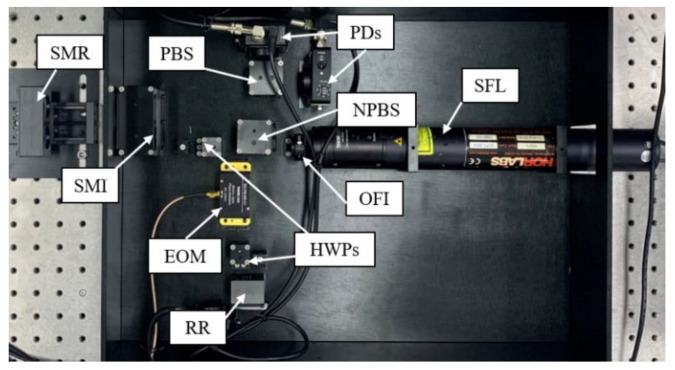
Experimental setup of the SPMAI.

In the experiments, an SPMAI prototype was constructed and developed into a measurement instrument, as shown in Figure 7. From Figure 7, one can see that the whole optical structure was installed within a metal box. The SFL (HRS015, Thorlabs), OFI (IO-2D-633-VLP, Thorlabs), EOM (EO-PM-NR-C1, Thorlabs), PDs (PDA36A2, Thorlabs) and other optical elements (PBSs, BSs, NPBSs and retro-reflectors) were used in the experiments. The typical wavelength of the SFL was 632.991 nm with a frequency stability of ±0.004 ppm and its polarization ratio was higher than 1000. The EOM used was a DC-couple broadband version, which could provide a phase-modulation frequency ranging from DC to 100 MHz. Based on its specification, *V_π_* was 135 V at a wavelength of 633 nm for this EOM. The OFI was based on the Faraday effect, which can rotate the polarization direction of the input beam 45°. Two PDs were used to receive the measurement interference signals and convert the light signals into voltage signals. It is noted that the PDs used were amplifiable, switchable-gain, Silicon detectors and the gain of 20 dB was chosen to provide a bandwidth of 1 MHz. These PBSs, BSs, NPBSs and retro-reflectors were fixed by designed mechanical structures. The RR and SMR were composed of retro-reflectors with the same model (PS975-A, Thorlabs), the length of which was 22.0 mm and the diameter was 25.4 mm. Moreover, these retro-reflectors had a surface flatness of λ/10 at 633 nm and a clear aperture of 17.8 mm. For the SMR used, *d* was 31.21 mm. Meanwhile, these electrical and optical devices were fixed by several designed mechanical structures. In addition, a DAQ (USB-6212, NI) and a high voltage amplifier (HVA 200, Thorlabs) were adopted. The DAQ used had 16 analog input channels (AIs, 16-bit, 400 kS/s) and 2 analog output channels (AOs, 250 kS/s). The DAQ not only could collect the analog voltage signals from PDs, but could also output the analog voltage signals to modulate the EOM. In order to ensure that the EOM could provide enough modulation depth, the HVA was used to amplify the modulated voltage signals from the DAQ. Here, by means of the LabVIEW program for PC, the modulation frequency of sinusoidal voltage signals could be adjusted.

**Figure 8:**
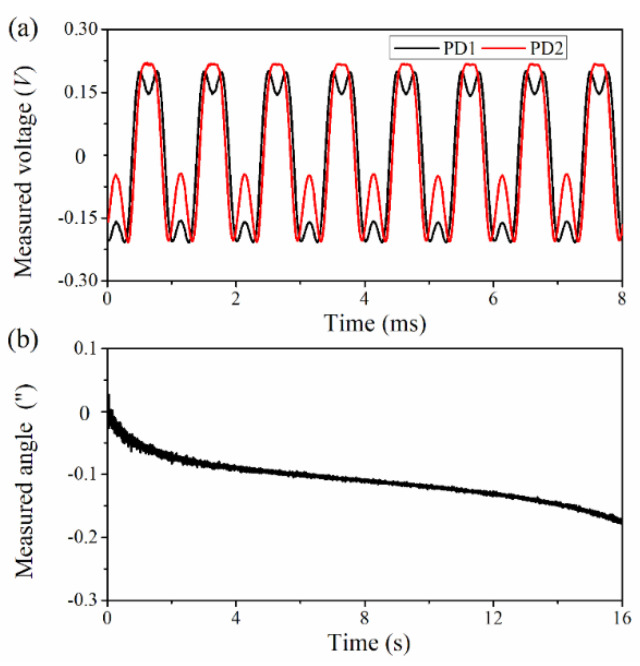
Static measured results of the SPMAI. (**a**) The measured interference signals when the SMR was still; (**b**) The angle variations over the measurement time range of 0–16 s.

Firstly, the static performance of the SPMAI was evaluated. Both the SMR and the SPMAI were placed on an optical platform and there was no applied noise suppression measure. Two PDs were connected to the AIs of the DAQ in a non-reference single ended (NRSE) manner, while one AO of the DAQ was connected to the HVA and the amplified signals was transmitted to the EOM. As the carrier frequency and sampling rate were respectively set to 10 kHz and 100 kHz, the interference signals were sampled by the DAQ and then transmitted to the PC. The amplitude of the carrier signals was adjusted to 5.58 V and the measured results are shown in Figure 8. Figure 8a presents the interference signals measured when the SMR was still, where the black line denotes the interference signals detected by the PD1 and the red line denoted the interference signals detected by the PD2. Using the PGC-Arctan algorithm on LabVIEW, the rotation angle of the SMR could be obtained. Figure 8b presents the angle variations over the measurement time range of 0–16 s. From Figure 8b, one can see that the angle values measured decreased from 0.027″ to −0.178″. Furthermore, the SPMAI had an average output angle value of −0.109″ with a standard deviation of 0.033″. These experimental results demonstrated that our proposed SPMAI had a good static performance.

**Figure 9:**
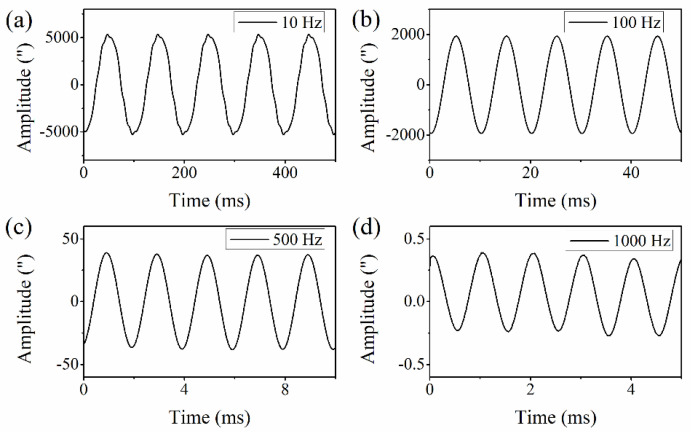
The measured results of the SPMAI at the angular vibration frequencies of (**a**) 10 Hz, (**b**) 100 Hz, (**c**) 500 Hz and (**d**) 1000 Hz.

Next, a high-frequency angular vibration exciter system was established to evaluate the dynamic performance of the SPMAI. Referring to [19], the angular vibration exciter system used comprised a signal generator (XF1965, NF), a power amplifier (ATA-3040, Aigtek) and an angular vibration table. The signal generator produced a sinusoidal electrical signal, the amplitude and frequency of which could be adjusted. This electrical signal was amplified by a power amplifier and transmitted into an angular vibration table. The signal generator used could provide a tunable frequency ranging from 0.01 μHz to 15 MHz, with a frequency accuracy of 0.01 μHz. The power amplifier used could work well within a frequency range of DC–30 kHz with a slew rate of 10 V/μs and its maximal output power reached 360 W. In addition, the used angular vibration table was specially designed and fabricated by our lab and was driven by a brushless DC motor. The SMR was fixed on the top of the angular vibration table. As the electrical signals from the signal generator drove the angular vibration table, the SMR was rotated a certain angle and thus the interference signals of the SPMAI showed a corresponding change. According its specifications, the high-frequency angular vibration exciter system used could provide an angular vibration within the frequency range of 1–1000 Hz. In the experiments, the real-time rotation angle values were measured as the angular vibration frequencies were increased from 1 Hz to 1000 Hz with an arbitrary increment. It should be noted that the amplitude of the angular vibration table used was adjustable. Figure 9 presents the results measured with the angular vibration frequencies of 10 Hz, 100 Hz, 500 Hz and 1000 Hz. From these results, one can infer that the measured amplitude was decreased from 5444.60″ at a frequency of 10 Hz to 0.31″ at a frequency of 1000 Hz, which was mainly limited by the angular acceleration of the angular vibration table. The measured results in Figure 9a–d show that there exists a steady amplitude in several angular vibration cycles. Furthermore, the amplitude values measured at several typical angular vibration frequencies are presented in Table 4. From the experimental results above, one can find that our proposed SPMAI can realize angular vibration measurement with an amplitude of sub-arcsecond within the frequency range of 1–1000 Hz.

**Figure 10:**
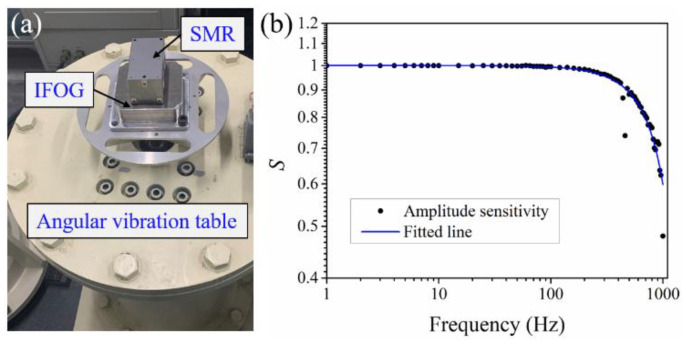
(**a**) Experimental setup for measuring bandwidth of the IFOG using the SPMAI; (**b**) Relationship between the amplitude sensitivity and the angular vibration frequency.

Finally, the proposed SPMAI was used to measure the bandwidth of an IFOG by simultaneously measuring the real-time rotation angle. The experimental setup is shown in Figure 10a. The IFOG and the AMR were fixed on top of the angular vibration table. The IFOG is a well-known ISD, which can directly measure the angular velocity. Here, the used IFOG was fabricated by our lab and had a diameter of 70 mm and a sampling rate of 10 kHz. We employed the serial port to read the output data of the IFOG and the rotation angle of the angular vibration table could be obtained by accumulating the output of the IFOG. As the angular vibration was adjusted from 1 Hz to 1000 Hz, the real-time angle values of the angular vibration table were measured and, furthermore, their corresponding amplitudes were obtained. By comparing the amplitude values measured by the IFOG and the SPMAI, the amplitude sensitivity *S* were calculated as

(17) $$S=\frac{A_{ifog}}{A_{spmai}},$$

where *A_ifog_* is the measured amplitude value of the IFOG, while *A_spmai_* is the measured amplitude value of the SPMAI. The variation of the amplitude sensitivity relative to the angular frequencies is shown as Figure 10b. From Figure 10b, one can see that the amplitude values measured by the SPMAI and the IFOG showed a slightly difference when the angular vibration frequency was less than 200 Hz. With the increase in the angular vibration frequency, *A_ifog_* decreased significantly relative to *A_spmai_*. From the experimental results, the 3 dB bandwidth of the IFOG was be calculated to be 848 Hz. In addition, the measurement uncertainty of the SPMAI in calibrating IFOG is also discussed. According to the Guide to the Expression of Uncertainty in Measurements (GUM) [20], the measurement uncertainty may come from the measurement instruments used, measurement principle, measurement environment and operation process. From Equation (18), one can infer that the measurement uncertainty of the amplitude sensitivity is related to the SPMAI and the IFOG. Referring to the GUM, the measurement uncertainty model of the amplitude sensitivity can be established as

(18) $$\delta S=\frac{\delta A_{ifog}}{A_{spmai}}-\frac{\delta A_{spmai}}{A_{spmai}^{2}}A_{ifog},$$

where *δ A_ifog_* denotes the measurement uncertainty of the IFOG, while *δ A_spmai_* denotes the measurement uncertainty of the SPMAI. Based on the theoretical and experimental results above, the measurement uncertainty budget is shown in Table 5. It should be noted that the angular vibration frequency of 100 Hz was chosen, at which the measured amplitude of the SPMAI was 1948.58″ and the measured amplitude of the IFOG was 1939.53″. From the results in Table 5, we can calculate the expanded uncertainty of the amplitude sensitivity as 0.8% at the angular vibration frequency of 100 Hz, as the coverage factor is 2.

## 5. Conclusions

In this paper, we demonstrate an angle interferometer based on sinusoidal phase modulation used for high-frequency angular vibration measurements. The optical configuration, angle measurement principle and PGC demodulation algorithm are described in detail. Several error factors in the PGC-Arctan demodulation algorithm for angular vibration measurements are analyzed, including the modulation depth deviation, carrier phase delay and carrier modulation frequency. Both theoretical and experimental results demonstrated that our proposed SPMAI can realize an angular vibration measurement over the frequency range of 1–1000 Hz. Finally, the proposed SPMAI can be used for calibrating ISDs, such as an IFOG.

## Figures and Tables

**Table 1:** The calculated THD and SINAD with different *c_m_*.

*c_m_* (Rad)	Amplitude (″)	THD (%)	SINAD (dB)
1.63	1.002	14.94	16.47
2.03	1.001	9.42	20.43
2.63	1.002	0.33	56.22
3.03	1.002	8.93	21.18
3.63	1.004	41.77	7.60

**Table 2:** The calculated THD and SINAD with different *c_m_*.

*σ* (°)	Amplitude (″)	THD (%)	SINAD (dB)
0	1.002	0.34	57.27
20	1.003	4.17	27.30
40	1.002	25.63	11.76
60	1.000	0.39	55.94
80	1.005	42.61	7.47

**Table 3:** The calculated THD and SINAD with different *c_m_*.

D (″)	Amplitude (″)	THD (%)	SINAD (dB)
0.6	0.601	0.50	47.36
1.2	1.212	1.67	35.55
1.8	1.859	3.80	28.31
2.4	2.425	6.62	23.41
3	3.000	10.46	19.24

**Table 4:** The measured results of the SPMAI at different angular vibration frequencies.

Frequency	Amplitude (″)	Frequency	Amplitude (″)
1 Hz	5526.43	100 Hz	1948.59
5 Hz	6408.53	300 Hz	63.48
10 Hz	5444.60	500 Hz	37.52
50 Hz	1907.51	800 Hz	1.04
80 Hz	4275.08	1000 Hz	0.31

**Table 5:** The measurement uncertainty budget of the amplitude sensitivity of the IFOG.

Sources of Uncertainty		Specification	Uncertainty
SPMAI	Short-term repeatability	0.033″	0.033″
	Separation distance of AMR	0.0009 mm	0.063″
	Thermal expansion of AMR	1.5 ppm	0.003″
	Laser wavelength stability	0.02 ppm	0.001″
	Refractive index variation	1.8 ppm	0.004″
	Demodulation algorithm	0.08″	0.08″
IFOG	Scale factor repeatability	346.93 ppm	0.673″
	Combined standard uncertainty *u*(S)		0.4%
	Expanded uncertainty (k = 2) *U*(S)		0.8%

## Data Availability

Not applicable.
